# Group II mGlu receptor antagonist LY341495 enhances the antidepressant-like effects of ketamine in the forced swim test in rats

**DOI:** 10.1007/s00213-016-4325-7

**Published:** 2016-06-11

**Authors:** Karolina Podkowa, Bartłomiej Pochwat, Piotr Brański, Andrzej Pilc, Agnieszka Pałucha-Poniewiera

**Affiliations:** Department of Neurobiology, Institute of Pharmacology Polish Academy of Sciences, Smętna 12, 31-343 Kraków, Poland; Department of Drug Management, Faculty of Health Sciences, Jagiellonian University Medical College, Grzegórzecka 20, 31-531 Kraków, Poland

**Keywords:** Antidepressant, Depression, Forced swim test, Ketamine, LY341495, Metabotropic glutamate receptor, mTOR

## Abstract

**Rationale:**

Numerous preclinical and clinical studies have reported the rapid and sustained antidepressant effects of the NMDA receptor antagonist ketamine. Because ketamine induces several undesirable and dangerous effects, a variety of strategies have been suggested to avoid such effects.

**Objectives:**

Here, we propose to enhance the sub-effective doses of ketamine by co-administration with the group II metabotropic glutamate (mGlu) receptor antagonist LY341495. This compound potentially acts as an antidepressant via a mechanism similar to that of ketamine.

**Methods:**

To investigate the rapid and sustained antidepressant-like effects of these drugs, we administered ketamine and LY341495 individually or in combination, 40 min and 24 h before the forced swim test (FST).

**Results:**

We found that sub-effective doses of ketamine and LY341495, given jointly, induce significant antidepressant-like effects, at both 40 min and 24 h after administration. The results obtained using Western blot technique indicate that mammalian target of rapamycin (mTOR) pathway activation may be involved in the mechanism of this action. The effects of drugs, used at identical ranges of times and doses, on spontaneous locomotor activity in rats were excluded. Furthermore, the results obtained from the rota-rod test and the ketamine-induced hyperlocomotion test suggest a lack of potentially adverse effects from the combined administration of ketamine and LY341495 at doses previously used in the FST.

**Conclusion:**

Altogether, these data suggest that the joint administration of ketamine and LY341495 might be a noteworthy alternative to the use of solely ketamine in the therapy of depression.

## Introduction

The current treatment of depression is based on the modulation of biogenic amine systems and requires the long-term administration (3–6 weeks) of antidepressant drugs (ADs) in order to obtain positive therapeutic effects (Millan 2006). On the other hand, several clinical studies have shown that sub-anesthetic doses of a noncompetitive NMDA antagonist, ketamine, can induce fast relief of depressive symptoms, which lasts for a few days or a week in patients affected by major depressive disorder (MDD) (Berman et al. 2000; Phelps et al. 2009; Rasmussen et al. 2013; Zarate et al. 2006). Accumulating evidence also points to the rapid and sustained antidepressant-like activity of ketamine in rodent behavioral models of depression, including the forced swim test (FST), the tail suspension test (TST), the learned helplessness paradigm, and chronic mild stress (CMS) (Garcia et al. 2008, 2009; Gigliucci et al. 2013; Koike et al. 2011; Li et al. 2010; Maeng et al. 2008). One of the leading hypotheses to explain the unique antidepressant properties of ketamine suggests that ketamine affects extracellular glutamate release, resulting in the enhancement of AMPA receptor activation and the subsequent increase in the release of BDNF. Consequently, BDNF stimulates TrkB receptors and leads to fast activation of the mammalian target of rapamycin (mTOR) signaling pathway, resulting in the augmentation of the synaptogenesis in the prefrontal cortex (PFC) (Duman et al. 2012; Li et al. 2010). According to this hypothesis, enhanced synaptogenesis as a result of the activation of the mTOR cascade appears to be a key mechanism of the rapid and sustained antidepressant action of ketamine. Interestingly, several studies have shown that functional synaptic changes in the PFC, which appear 24 h after ketamine administration, correlate with the antidepressant effects of the drug in animal models of depression (Koike et al. 2011; Li et al. 2010; Maeng et al. 2008).

Thus, it appears that ketamine could become a breakthrough treatment for depression. However, we note that the use of ketamine can lead to dangerous consequences, including dissociative and pro-psychotic side effects. Moreover, due to its hallucinogenic properties, ketamine may be overused (Pal et al. 2002). In addition, ketamine not only stimulates psychosis in patients suffering from schizophrenia but also induces psychosis-like states in previously healthy patients, which limits its usage as an AD for outpatients (Krystal et al. 1998; Lathi et al. 1995; Malhotra et al. 1996). To overcome these limitations, several new pharmacological strategies have been proposed, including enhancement of the therapeutic effects of ketamine by combining its administration with other antidepressant agents, e.g., lithium (Chiu et al. 2014; Liu et al. 2013).

Numerous studies have shown that antagonists of group II metabotropic glutamate (mGlu) receptors (including mGlu2 and mGlu3 subtypes), MGS0039 and LY341495, evoke certain effects that resemble the effects of ketamine. For example, these drugs induce rapid and prolonged antidepressant-like effects in the FST and the TST (Chaki et al. 2004; Koike and Chaki 2014; Pałucha-Poniewiera et al. 2010, 2014), and the mechanism of their antidepressant action is related to AMPA receptor stimulation, activation of the mTOR pathway, and the synthesis of synaptic proteins in the PFC (Dwyer et al. 2012; Karasawa et al. 2005; Koike et al. 2011; Koike and Chaki 2014). In contrast, behavioral studies suggest that group II mGlu receptor antagonists will not induce serious adverse effects that are typical of ketamine. Because the unique actions of ketamine and the group II mGlu receptor antagonist might both be associated with stimulation of the same molecular pathways, we hypothesized that LY341495 could enhance the antidepressant-like effects of ketamine, while undesirable ketamine-induced effects will not be induced.

Therefore, in the present study, we investigated whether joint administration of low, sub-effective doses of ketamine and LY341495 exhibit both acute (40 min after single injection) and prolonged (24 h after single injection) antidepressant-like activity in the FST of rats. The second aim of this experiment was to assess a profile of the potential adverse effects of this combination. The last goal of the study was to investigate if mTOR pathway and synaptic protein synthesis might be involved in the mechanism of action of a combined administration of ketamine and LY341495.

## Materials and methods

### Animals and housing

Male Sprague-Dawley rats (weighing 250–350 g) obtained from Charles River Laboratories, Germany were used in this study. The animals were housed five per cage and maintained under standard laboratory conditions of temperature (19–21 °C) and lighting (light phase 6 a.m.–6 p.m.). Food and water were available ad libitum. Each experimental group consisted of seven to ten animals, and each rat was used only once. Behavioral experiments were performed between 9 a.m. and 2 p.m. by an experimenter unaware of the treatment. All procedures were carried out in accordance with the criteria of the National Institutes of Health Animal Care and Use Committee and were approved by the Second Local Ethics Committee in Krakow, at the Institute of Pharmacology, Polish Academy of Sciences.

### Drug administration

Ketamine hydrochloride solution (Bioketan, Biowet, Poland) was diluted with 0.9 % NaCl, while LY341495 (Tocris Cookson Ltd., Bristol, UK) was dispersed in a suspension of 0.5 % methylcellulose using an ultrasonic homogenizer (Core-Parmer Instrument Co., Chicago, IL, USA). Control groups received 0.9 % NaCl or 0.5 % methylcellulose. All chemicals were administered intraperitoneally (IP) at a constant volume of 1 ml/kg body weight.

### The forced swim test

Two sessions of the experiment were performed according to the procedure described by Porsolt et al. (1978), with some necessary modifications. On the first day of the experiment, rats were placed individually in glass cylinders (40 cm high, 18 cm in diameter) containing 25 cm of water that was maintained at 25 °C. The water column was sufficiently deep so that the rats could not support themselves by placing their paws on the base of the cylinder. After 15 min, the rats were placed in a drying room (30 °C) for 30 min. The rats were placed again in the cylinder 24.5 h later, and the total duration of immobility was measured during the 5-min main test. The following three different behaviors were rated: (1) immobility—rats were considered to be immobile when they remained passively floating in the water; (2) swimming—rats were considered to be swimming if they were making active swimming motions, more than necessary to solely maintain their head above water; and (3) climbing—rats were considered to be climbing when they were making active movements in and out of the water with their forepaws, usually directed against the walls. Each parameter in the FST was measured manually by an independent experimenter unaware of the treatment. Ketamine, LY341495, a mixture of ketamine and LY341495, or vehicle was administered IP 40 min or 24 h before the main test.

### Spontaneous locomotor activity

Spontaneous locomotor activity was recorded individually for each animal in Opto-Varimex (Columbus Instruments, Columbus, OH, USA) plexiglass chambers (43 × 44 × 25 cm) connected to a compatible PC. Each cage was surrounded by a 15 × 15 array of photocell beams located 3 cm from the floor surface. Interruptions of the photobeams were interpreted as horizontal activity, and the distance traveled was converted into centimeters. All the data were analyzed using Auto-track software (Columbus Instruments, USA). The rats were administered IP injections of ketamine, LY341495, a combination of the two drugs, or vehicle in their home cages: 40 min or 24 h after drug administration, the rats were placed individually into the clean locomotor activity chambers. Immediately thereafter, locomotor activity was measured every 5 min for 30 min.

### Ketamine-induced hyperactivity

Ketamine-induced hyperactivity was measured using the equipment previously used for evaluation of the exploratory activity of rats. Rats were placed individually into actometers for an acclimation period of 60 min. Then, they were administered an IP injection of ketamine, LY341495, a combination of the two compounds, or vehicle. Immediately after injection, each rat was returned into the chamber. From then on, the total distance traveled was measured within a 60-min experimental session and stored every 10 min.

### Rota-rod test

The rota-rod test was performed in order to assess ketamine-induced impairment of motor coordination. The procedure was performed according to Vogel et al. (2008) with some modifications. The rats were placed on the rota-rod apparatus (ENV-577; Med Associates, St. Albans, VT) rotating at a constant speed of 6 revolutions per minute (rpm). Three experimental sessions were performed. During the first session, the animals were individually placed on a rotating rod for 2 min. If the rat fell off of the rod, it was placed on the rod again. The second session, lasting 2 min, was performed 10 min after the first session. During this session, each rat that fell off at least twice was excluded from the experiment. The third experimental session was carried out 2 h later. LY341495 was administered 20 min before the test, whereas ketamine was given 2 min before the test. The same schedule of treatment was adopted when a combination of the two drugs was administered. The total number of animals that fell off before the end of this session was recorded and the latency to fall (expressed in s) was measured.

### Synaptosome preparation and Western blotting

Tissue samples were dissected from the PFC and hippocampus and homogenized in ice-cold lysis buffer [0.32 M sucrose, 20 mM HEPES (pH 7.4); 1 mM EDTA; 1× protease inhibitor cocktail, 5 mM NaF, and 1 mM NaVO_3_]. Homogenates were than centrifuged at 2800 rpm for 10 min at 4 °C. The resulting supernatant was then centrifuged at 12,000 rpm for 10 min at 4 °C. The resulting pellets were sonicated in protein lysis buffer containing 50 mM Tris-HCl (pH 7.5), 150 mM NaCl, 1 % Triton X-100, 0.1 % SDS, 2 mM EDTA, 1 mM NaVO_3_, 5 mM NaF, and protease inhibitor cocktail. Protein concentrations were measured using a BCA kit (Thermo Scientific, USA). Next, the proteins were separated by SDS-PAGE and transferred to nitrocellulose membranes and blocked for 1 h in 1 % of blocking solution [BM Chemiluminescence Western Blotting Kit (Mouse/Rabbit) made by Roche, Switzerland]. After blocking, the membranes were incubated overnight at 4 °C with the primary antibodies. The following antibodies were used: mTOR (1:500), phospho-mTOR (pmTOR, Ser2448, 1:500), p70S6K (1:500), phospho-p70S6K (pp70S6K, Thr389, 1:200), GluA1 (1:1000), and PSD95 (1:1000)—all from Abcam, USA. On the following day, the membranes were washed three times for 10 min in Tris-buffered saline with Tween (TBS-T) and incubated for 30 min with anti-mouse/anti-rabbit-IgG-peroxidase-conjugated antibodies. This set of secondary antibodies was also a component of the BM Chemiluminescence Western Blotting Kit (Mouse/Rabbit) (Roche, Switzerland). After incubation, the membranes were washed three times for 10 min with TBS-T. In the last step, the blots were incubated with a detection reagent (Roche, Switzerland). The signal from the tested proteins was visualized and measured using a Fuji-Las 1000 system and Fuji Image Gauge v.4.0 software. To check the transfer and loading, β-actin was indicated on each blot. For this purpose, a primary monoclonal antibody (Millipore, Germany) was used. The final result is given as the ratio of the optical density of particular proteins to the optical density of β-actin.

### Statistics

Statistical analyses were performed using GraphPad Prism 5.01 (GraphPad Software, San Diego, CA, USA). All the results are expressed as the means ± SEM. Statistical significance was assessed using a one-way ANOVA followed by Dunnett’s post hoc test (to evaluate dose–effect curves in the FST) or a two-way ANOVA (when a drug combination was used in the FST and for the rota-rod test results). A repeated-measures ANOVA was used to analyze the locomotor activity results, and *t* test was used to analyze Western blotting data. The results were considered statistically significant if *p* < 0.05. More details concerning the statistical analysis are presented beneath the figures.

## Results

### The antidepressant-like effects of ketamine in the FST

**Fig. 1:**
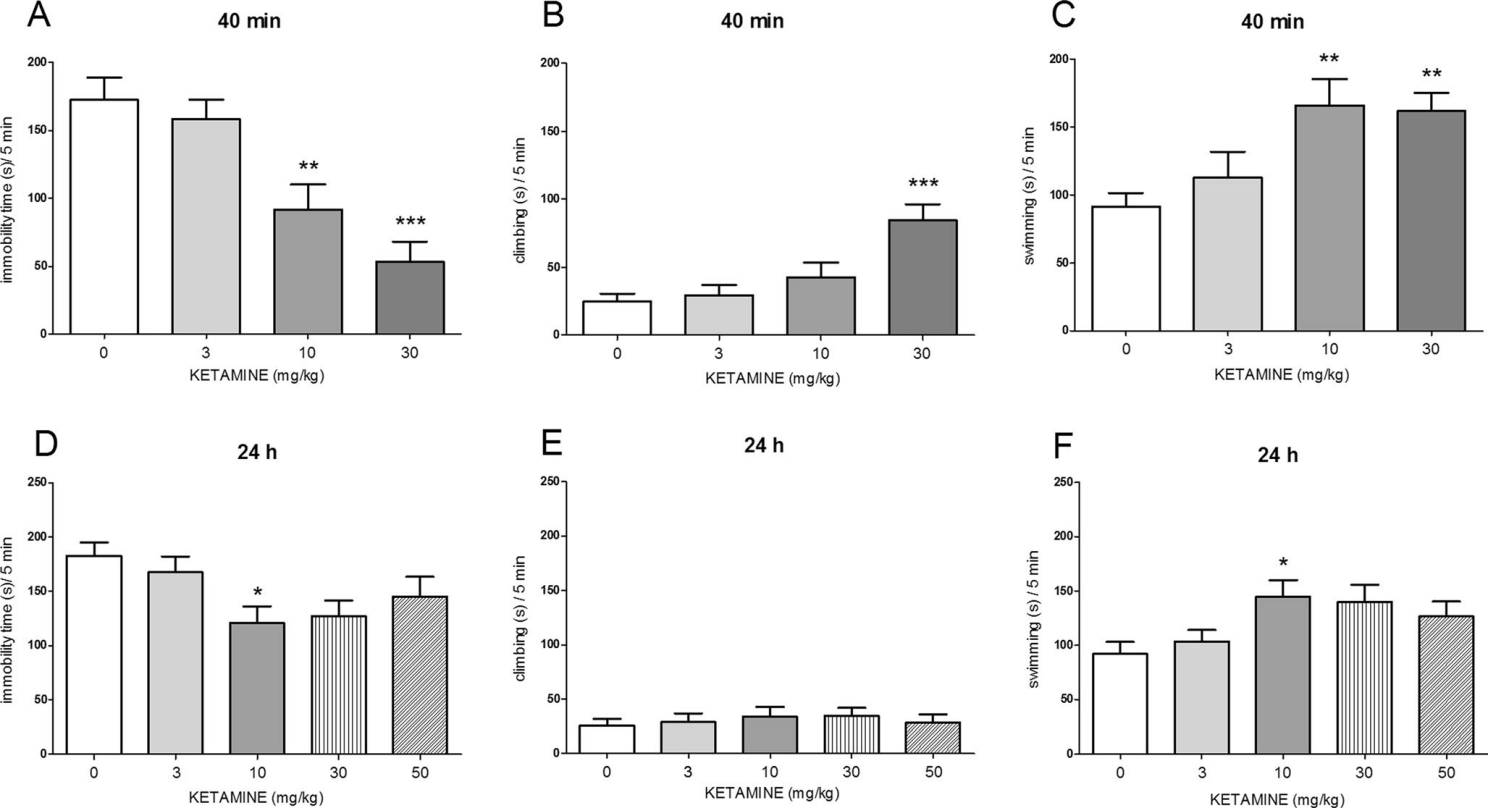
The antidepressant-like effect of ketamine administered IP 40 min before the FST (*upper panel*) or 24 h before the FST (*lower panel*) in rats. The following three parameters were measured: immobility (**a**, **d**), climbing (**b**, **e**), and swimming (**c**, **f**). Values are expressed as the means ± SEM and were analyzed by one-way ANOVA followed by Dunnett’s post hoc test (*p < 0.05; **p < 0.01; ***p < 0.001 vs. respective vehicle)

One-way ANOVA showed the effects of ketamine (3–30 mg/kg, IP), administered 40 min before the FST, on immobility time [*F*(3, 29) = 12.17, *p* < 0.0001; Fig. 1a], climbing time [*F*(3, 29) = 8.904, *p* < 0.001; Fig. 1b], and swimming time [*F*(3, 29) = 6.395, *p* < 0.01; Fig. 1c]. Post hoc tests showed that ketamine, given at a dose of 10 mg/kg, significantly (*p* < 0.01) changed the immobility time (Fig. 1a) and the swimming time (Fig. 1c), whereas at the dose of 30 mg/kg, it affected all three parameters: the immobility time (*p* < 0.001; Fig. 1a), the climbing time (*p* < 0.001; Fig. 1b), and the swimming time (*p* < 0.01; Fig. 1c). When applied at a dose of 3 mg/kg, ketamine did not affect any of the tested parameters.

Concerning the antidepressant-like activity of ketamine (3–50 mg/kg) administered 24 h before the FST, we observed the effect of this drug on immobility time [*F*(4, 33) = 2.941, *p* < 0.05; Fig. 1d] and swimming time [*F*(4, 33) = 2.899, *p* < 0.05; Fig. 1f], without any effect on climbing [*F*(4, 33) = 0.262, *p* > 0.05; Fig. 1e]. Post hoc analyzes showed that ketamine significantly decreased immobility time and increased swimming time only when it was applied at a dose of 10 mg/kg (*p* < 0.05; Fig. 1d, f).

### The antidepressant-like effects of the group II mGlu receptor antagonist LY341495 in the FST

**Fig. 2:**
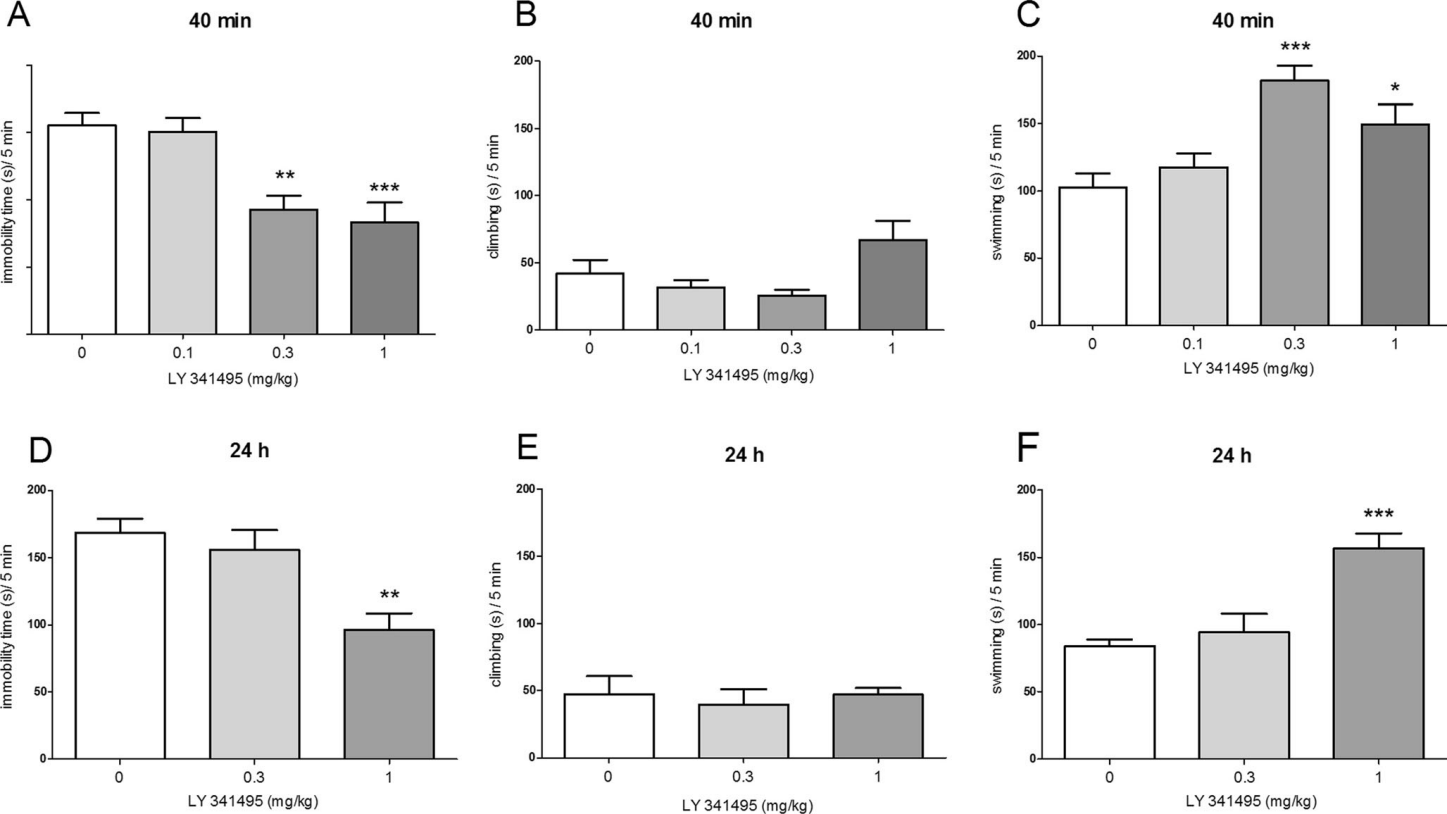
The antidepressant-like effect of LY341495 administered IP 40 min (*upper panel*) or 24 h (*lower panel*) before the FST in rats. The following three parameters were measured: immobility (**a**, **d**), climbing (**b**, **e**), and swimming (**c**, **f**). Values are expressed as the means ± SEM and were analyzed by one-way ANOVA followed by Dunnett’s post hoc test (*p < 0.05; **p < 0.01; ***p < 0.001 vs. respective vehicle)

A one-way ANOVA revealed that LY341495 (0.1–1 mg/kg, IP) administered 40 min before the FST significantly reduced immobility time [*F*(3, 33) = 10.98, *p* < 0.0001; Fig. 2a] and increased swimming time [*F*(3, 33) = 8.894, *p* < 0.001; Fig. 2c] but had no impact on climbing time [*F*(3, 33) = 1.499, *p* < 0.05; Fig. 2b]. The post hoc Dunnett test showed that LY341495 given at doses of 0.3 and 1 mg/kg significantly decreased the time of immobility (*p* < 0.01 and *p* < 0.001, respectively; Fig. 2a) and increased the time of swimming (*p* < 0.001 and *p* < 0.05, respectively; Fig. 2c). LY341495 given 40 min before the FST at the dose of 0.1 mg/kg failed to exert any effect.

When administered 24 h before the FST, LY341495 (0.3–1 mg/kg) significantly affected two parameters, immobility time [*F*(2, 20) = 8.741, *p* < 0.01; Fig. 2d] and swimming time [*F*(2, 20) = 13.76, *p* < 0.001; Fig. 2f], without any effect on climbing [*F*(2, 20) = 0.16, *p* > 0.05; Fig. 2e]. Post hoc analysis showed that LY341495 significantly decreased immobility (*p* < 0.01) and increased swimming time (*p* < 0.001) after injection of a 1-mg/kg dose, 24 h before the test. No changes were observed for this compound at the lower tested dose of 0.3 mg/kg, using the same schedule of administration.

### The effects of joint administration of ketamine and LY341495 in the FST

**Fig. 3:**
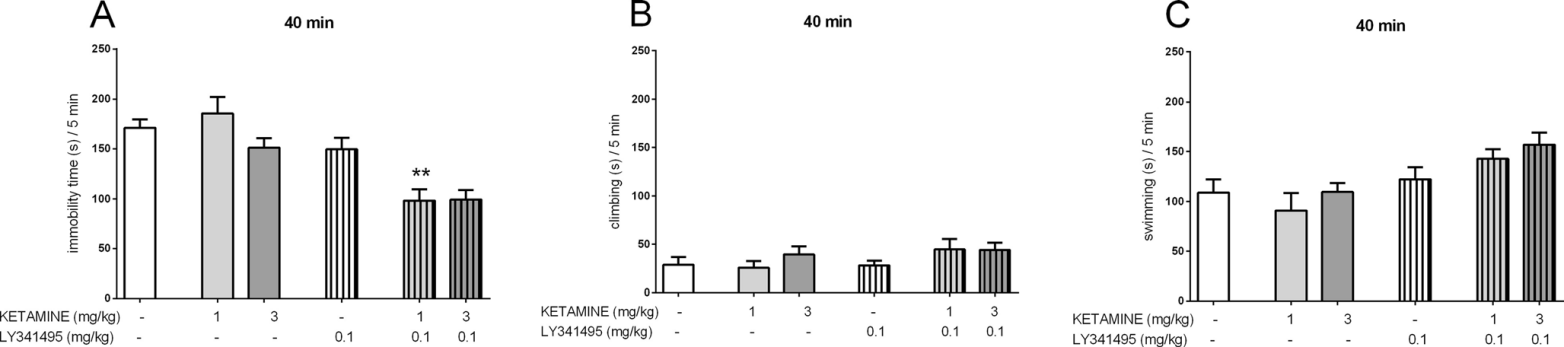
The antidepressant-like effect of joint administration of ketamine and LY341495 injected IP 40 min before the FST in rats. The following three parameters were measured: immobility (**a**), climbing (**b**), and swimming (**c**). Values are expressed as the means ± SEM and were analyzed by two-way ANOVA. **p < 0.01 interaction: ketamine (1 mg/kg) × LY341495 (0.1 mg/kg)

**Fig. 4:**
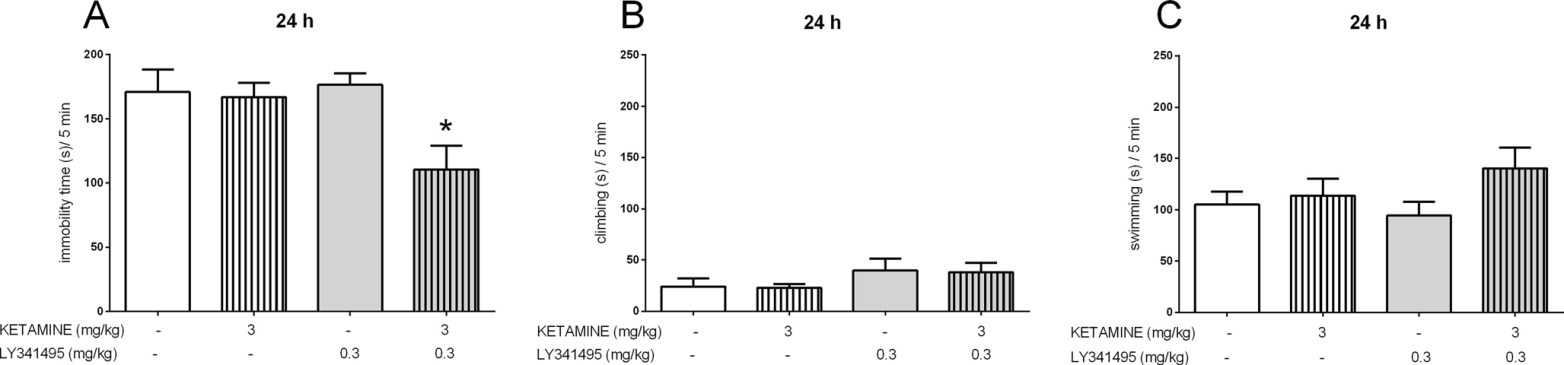
The antidepressant-like effect of joint administration of ketamine and LY341495 injected IP 24 h before the FST in rats. The following three parameters were measured: immobility (**a**), climbing (**b**), and swimming (**c**). Values are expressed as the means ± SEM and were analyzed by two-way ANOVA. *p < 0.05 interaction: ketamine (3 mg/kg) × LY341495 (0.3 mg/kg)

The joint administration of sub-effective doses of ketamine and LY341495 was used in the FST, and 40 min or 24 h after IP administration, we evaluated both rapid and sustained antidepressant effects. To study the rapid effect, the doses of 3 and 0.1 mg/kg, for ketamine and LY341495, respectively, were selected for the experiment. Four experimental groups were formed: a control group, ketamine (3 mg/kg) and LY341495 (0.1 mg/kg) groups, and a group given a mixture of ketamine and LY341495. A two-way ANOVA revealed a strong impact of LY341495 [*F*(1, 29) = 12.6, *p* < 0.01] and ketamine [*F*(1, 29) = 11.53, *p* < 0.01] on the immobility of mice. However, no interaction of the two parameters was observed [*F*(1, 29) = 2.138, *p* > 0.05], despite the apparent trend of decreased rat immobility by co-administration of the two substances (Fig. 3a). Because we did not observe a statistically significant interaction of both drugs, which could be due to the tendency of ketamine (3 mg/kg) to reduce immobility time, we decided to examine a lower dose of the drug (1 mg/kg) in combination with LY341495 (0.1 mg/kg). A two-way ANOVA analysis revealed an interaction between ketamine and LY341495 for the sub-effective doses [*F*(1, 27) = 7.947, *p <* 0.01], indicating a significant enhancement of the rapid effect of ketamine by LY341495 in the FST (Fig. 3a). Another experiment was designed to test prolonged antidepressant effects (24 h after a single injection) of sub-effective doses of ketamine and LY341495. A two-way ANOVA revealed that non-active doses of ketamine (3 mg/kg) and LY341495 (0.3 mg/kg), both administered 24 h before the FST, significantly decreased the immobility time of rats when administered jointly [*F*(1, 29) = 5.553, *p <* 0.05, Fig. 4a]. Such interaction indicates the enhancement of the sustained antidepressant-like effects of ketamine by LY341495. Separate analyses for the swimming time and climbing time results revealed no interactions between ketamine and LY341495. However, we did observe that joint administration 40 min before the test tended to increase the time of swimming (Fig. 3c).

### The effects of ketamine and LY341495 on the spontaneous locomotor activity of rats

**Fig. 5:**
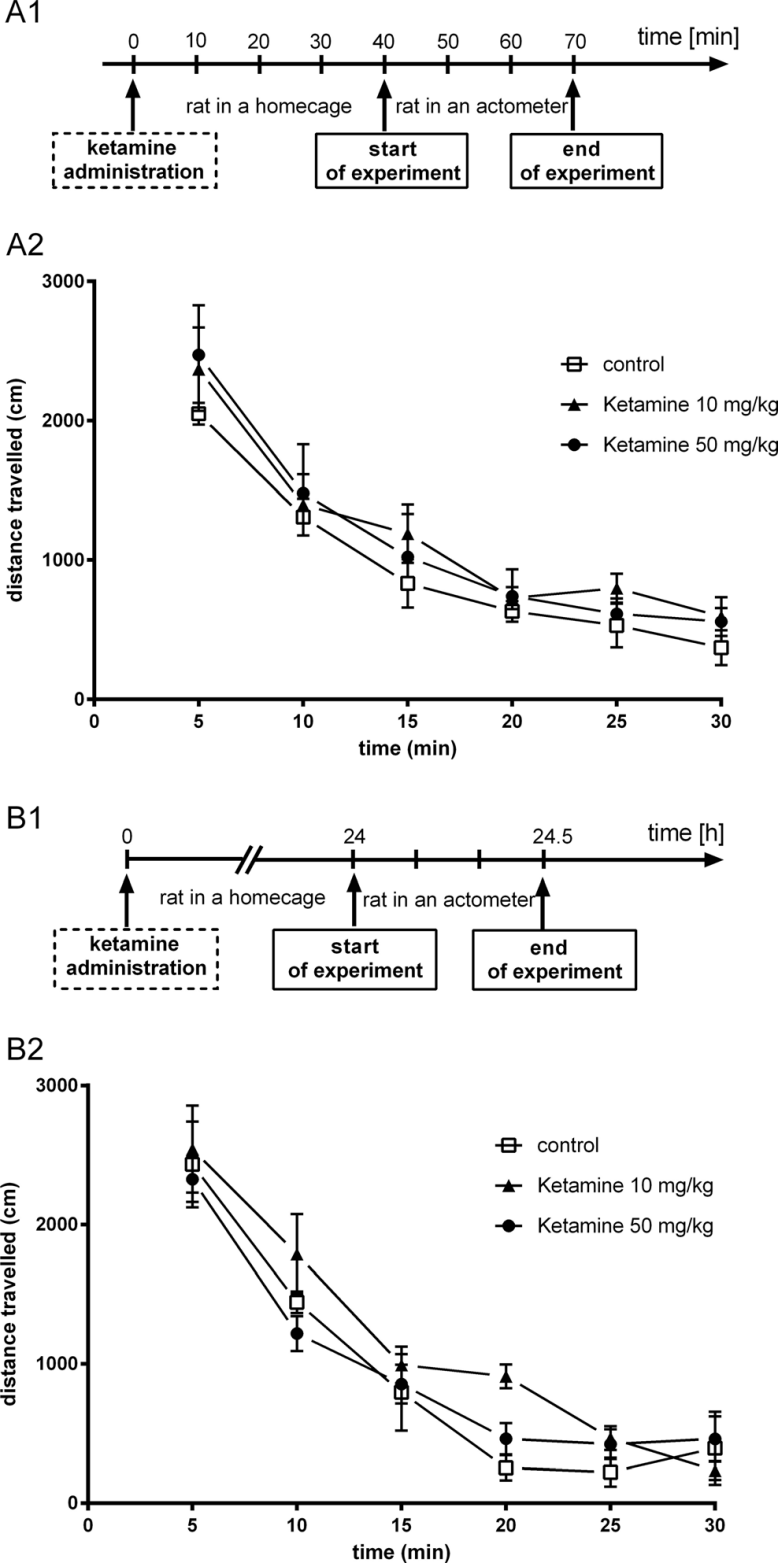
The effect of ketamine on the spontaneous locomotor activity of rats during a 30-min experimental session. Ketamine was administered IP 40 min (**a**) or 24 h (**b**) before the test. A1 and B1 represent schedules of the experimental procedures. A2 and B2 show the respective results. Values are expressed as the means ± SEM and were evaluated by repeated-measures ANOVA

**Fig. 6:**
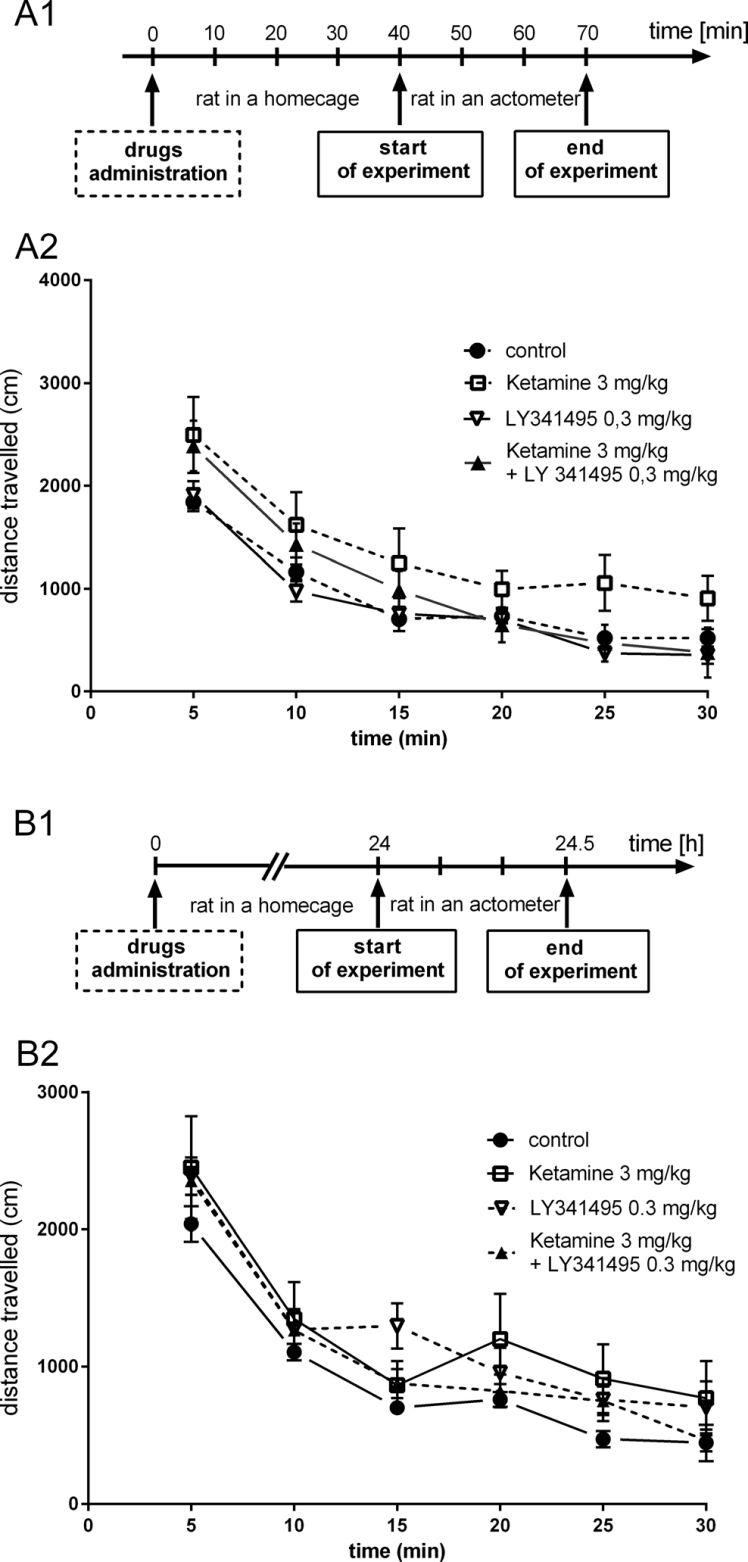
The effect of joint administration of ketamine and LY341495 on the spontaneous locomotor activity of rats during a 30-min experimental session. The compounds were administered IP 40 min (**a**) or 24 h (**b**) before the test. A1 and B1 represent schedules of the experimental procedures. A2 and B2 show the respective results. Values are expressed as the means ± SEM and were evaluated by repeated-measures ANOVA

Ketamine administered at doses of 10 or 50 mg/kg 40 min before the test did not significantly change the spontaneous locomotor activity of rats ([*F*(1, 11) = 1.191, *p* > 0.05] and [*F*(1, 11) = 0.412, *p* > 0.05], respectively; Fig. 5a). When we analyzed the influence of ketamine (10 or 50 mg/kg) injected 24 h before the locomotor activity test, we also noticed a lack of effect of this treatment on the distance traveled ([*F*(1, 10) = 2.14, *p* > 0.05] and [*F*(1, 10) = 0.06, *p* > 0.05], respectively; Fig. 5b). Another experiment aimed to measure the locomotor activity of rats treated with ketamine, LY341495, and the combination of these drugs, at the doses previously used in the FST, both 40 min and 24 h after injection. We found no significant changes between control rats and rats injected with ketamine (3 mg/kg) [*F*(1, 14) = 2.382, *p* > 0.05], LY341495 (0.3 mg/kg) [*F*(1, 14) = 0.685, *p* > 0.05], or the mixture of these drugs [*F*(1, 19) = 0.312, *p* > 0.05], when measured 40 min after injection (Fig. 6a). Similarly, no changes in the spontaneous locomotor activity of rats were observed 24 h after single administration of ketamine (3 mg/kg) [*F*(1, 12) = 2.842, *p* > 0.05], LY341495 (0.3 mg/kg) [*F*(1, 14) = 4.167, *p* > 0.05], or the combination of these compounds [*F*(1, 14) = 4.443, *p* > 0.05].

### The effect of joint administration of ketamine and LY341495 on the stimulation of the mTOR signaling pathway in the rat PFC and hippocampus

**Fig. 7:**
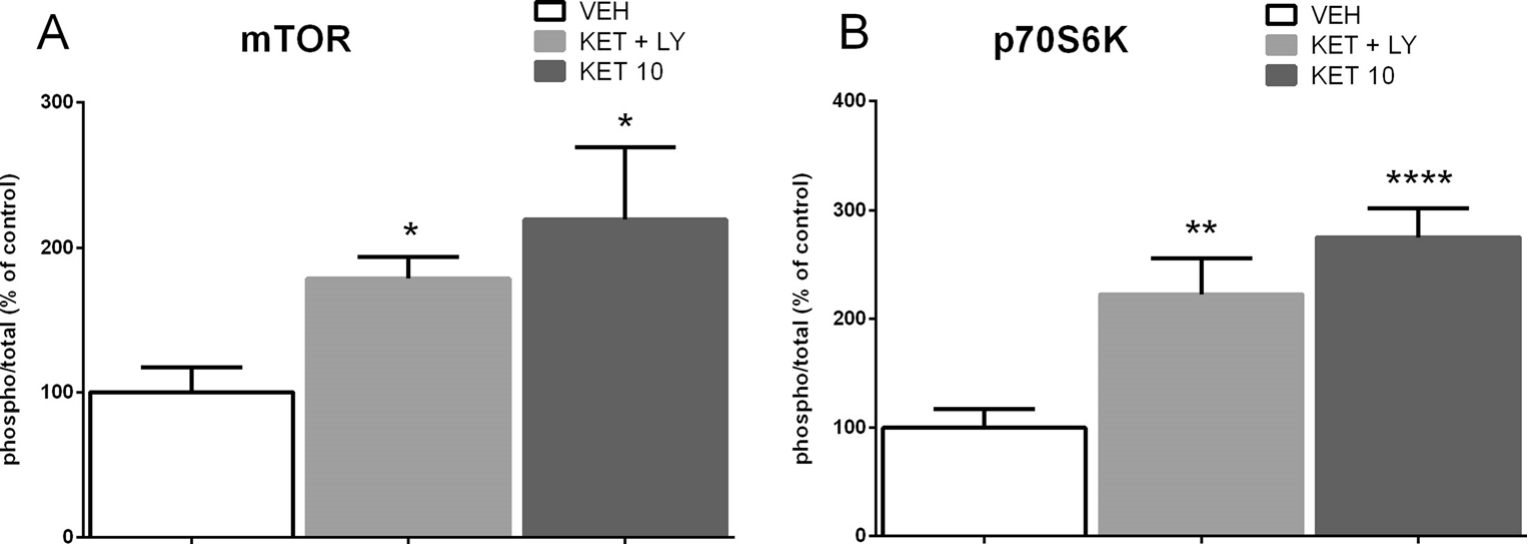
The effect of joint administration of ketamine (3 mg/kg) and LY341495 (0.3 mg/kg) on the stimulation of the mTOR signaling determined by Western blot analysis of pmTOR, mTOR, pp70S6K, and p70S6K in the synaptosome-enriched fraction of the rat PFC. The density of the obtained bands for phosphorylated and total forms of the studied proteins were first normalized to β-actin bands, and then, the ratio of the normalized phospho/total forms was calculated for each protein. The data were analyzed by *t* test comparing the expression values between vehicle-treated group (*VEH*) and ketamine (3 mg/kg) + LY341495 (0.3 mg/kg)-treated group (*KET + LY*) or ketamine (10 mg/kg)-treated group (*KET 10*). Values (the means ± SEM) are expressed as percentage of changes vs. control levels (*n* = 8; *p < 0.05, **p < 0.01, ****p < 0.0001 vs. vehicle)

**Fig. 8:**
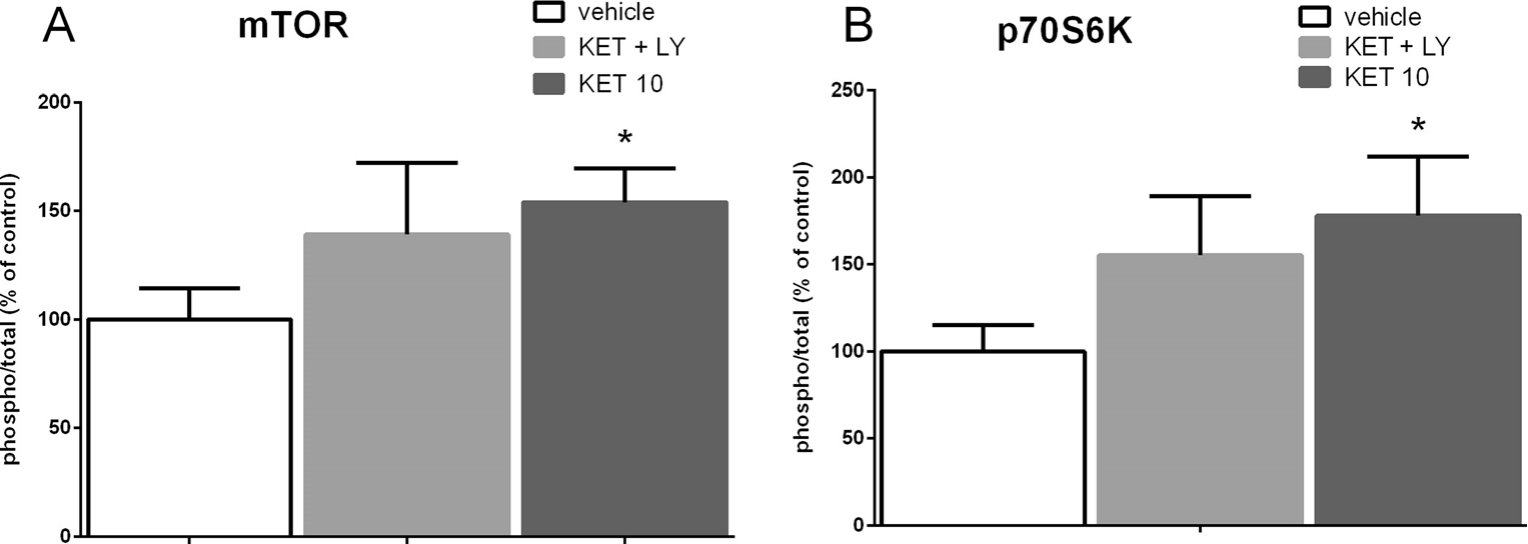
The effect of joint administration of ketamine (3 mg/kg) and LY341495 (0.3 mg/kg) on the stimulation of the mTOR signaling determined by Western blot analysis of pmTOR, mTOR, pp70S6K, and p70S6K in the synaptosome-enriched fraction of rat hippocampus. The density of the obtained bands for phosphorylated and total forms of the studied proteins were first normalized to β-actin bands, and then, the ratio of the normalized phospho/total forms was calculated for each protein. The data were analyzed by *t* test comparing the expression values between vehicle-treated group (*VEH*) and ketamine (3 mg/kg) + LY341495 (0.3 mg/kg)-treated group (*KET + LY*) or ketamine (10 mg/kg)-treated group (*KET 10*). Values (the means ± SEM) are expressed as percentage of changes vs. control levels (*n* = 8; *p < 0.05 vs. vehicle)

To investigate whether ketamine and LY341495, given jointly at doses that induced a sustained antidepressant-like effect in rats, stimulate mTOR signaling, we examined the influence of tested compounds on the total and phosphorylated forms of mTOR and p70S6K in the synaptosome-enriched fraction of the PFC 40 min or 24 h after injection. Ketamine at a dose of 10 mg/kg was used as a positive reference group. The density of the obtained bands for phosphorylated and total forms of the studied proteins were first normalized to β-actin bands, and then, the ratio of the normalized phospho/total forms was calculated for each protein. Statistical analysis of Western blot results in the PFC showed that ketamine (3 mg/kg) and LY341495 (0.3 mg/kg) given jointly 40 min before decapitation induced a significant increase in the phosphorylated forms of mTOR (*p* < 0.05) and p70S6K (*p* < 0.01) compared with a control group, by 178 and 222 %, respectively (Fig. 7a, b). The similar effect was produced by ketamine (10 mg/kg) which induced a significant increase in both pmTOR (*p* < 0.05) and pp70S6K (*p* < 0.0001), by 219 and 275 %, respectively (Fig. 7a, b). The similar but weaker effects were observed in the hippocampus, where a mixture of ketamine (3 mg/kg) and LY341495 (0.3 mg/kg) induced a noticeable but not statistically significant increase in the level of phosphorylated forms of both mTOR (*p* > 0.05) and p70S6K (*p* > 0.05), by 139 and 155 %, respectively (Fig. 8a, b). At the same time, ketamine (10 mg/kg) produced a significant increase in the phosphorylated forms of both mTOR (*p* < 0.05) and p70S6K (*p* < 0.05), by 154 and 178 % (Fig. 8a, b).

### The effect of joint administration of ketamine and LY341495 on the synaptic protein expression in the rat PFC and hippocampus

**Fig. 9:**
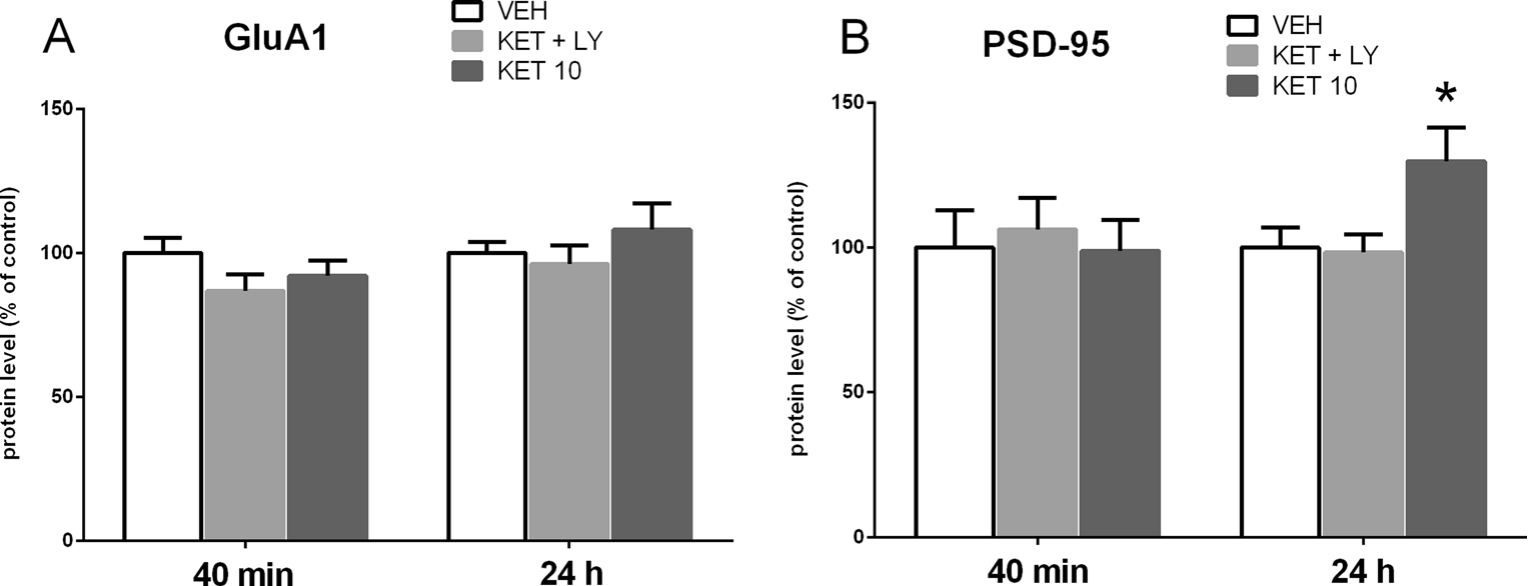
The effect of joint administration of ketamine (3 mg/kg) and LY341495 (0.3 mg/kg) on the expression of synaptic proteins: GluA1 (**a**) and PSD-95 (**b**) in the rat PFC. The density of the obtained bands of the studied proteins was normalized to β-actin bands. The data were analyzed by *t* test comparing the expression values between vehicle-treated group (*VEH*) and ketamine (3 mg/kg) + LY341495 (0.3 mg/kg)-treated group (*KET + LY*) or ketamine (10 mg/kg)-treated group (KET 10). Values (the means ± SEM) are expressed as percentage of changes vs. control levels (*n* = 8; *p < 0.05 vs. vehicle)

**Fig. 10:**
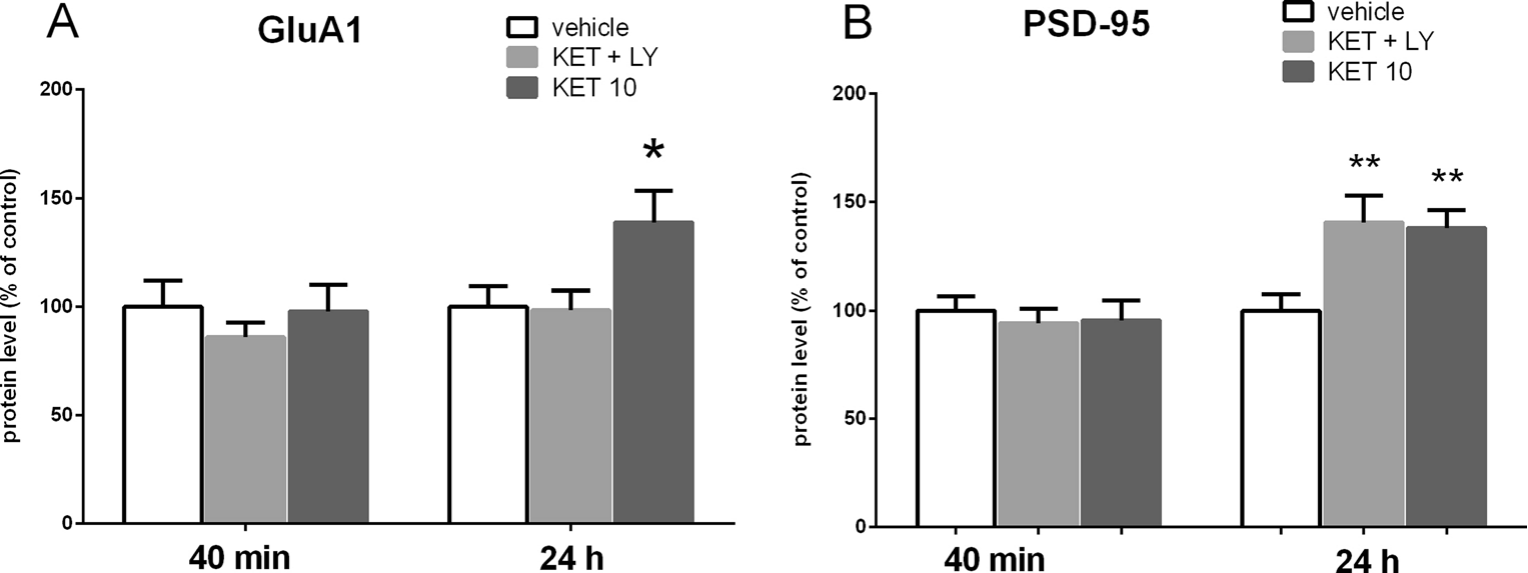
The effect of joint administration of ketamine (3 mg/kg) and LY341495 (0.3 mg/kg) on the expression of synaptic proteins: GluA1 (**a**) and PSD-95 (**b**) in rat hippocampus. The density of the obtained bands of the studied proteins was normalized to β-actin bands. The data were analyzed by *t* test comparing the expression values between vehicle-treated group (*VEH*) and ketamine (3 mg/kg) + LY341495 (0.3 mg/kg)-treated group (*KET + LY*) or ketamine (10 mg/kg)-treated group (*KET 10*). Values (the means ± SEM) are expressed as percentage of changes vs. control levels (*n* = 8; *p < 0.05, **p < 0.01 vs. vehicle)

To determine whether a mixture of ketamine and LY341495, used at the doses that had previously resulted in a positive effect in the FST, influence synaptic protein expression in the PFC and hippocampus, we investigated the influence of the tested compounds on the expression levels of GluA1 and PSD-95 in the synaptosome-enriched fraction of the PFC or hippocampus. Western blot analysis showed no effect of a mixture of ketamine and LY341495 on the expression level of GluA1 protein in the PFC both 40 min and 24 h after administration of drugs (*p* > 0.05; Fig. 9a). Ketamine used at a dose of 10 mg/kg did not also induce any effect on the expression level of GluA1, both 40 min and 24 h after administration (Fig. 9a). However, a statistically significant increase in the expression level of PSD-95 was observed in the PFC in a group of rats pre-treated with ketamine (10 mg/kg), 24 h after administration (*p* < 0.05; Fig. 9b). At the same time, a combined administration of ketamine and LY341495 did not induce any changes in the expression level of PSD-95 in any tested time point (40 min or 24 h after administration; Fig 9b). In contrast, we found a significant increase in hippocampal levels of both GluA1 (*p* < 0.05) and PSD-95 (*p* < 0.01), by 139 and 138 % respectively, 24 h after ketamine (10 mg/kg) administration and no changes in the level of both GluA1 and PSD-95 40 min after ketamine injection (*p* > 0.05; Fig. 10a, b). A mixture of ketamine and LY341495 induced a significant increase in the level of PSD-95 (*p* < 0.01) by 138 %, while it did not influence the hippocampal level of GluA1, both 40 min and 24 h after administration (*p* > 0.05; Fig. 10a).

### Ketamine-induced hyperlocomotion test

**Fig. 11:**
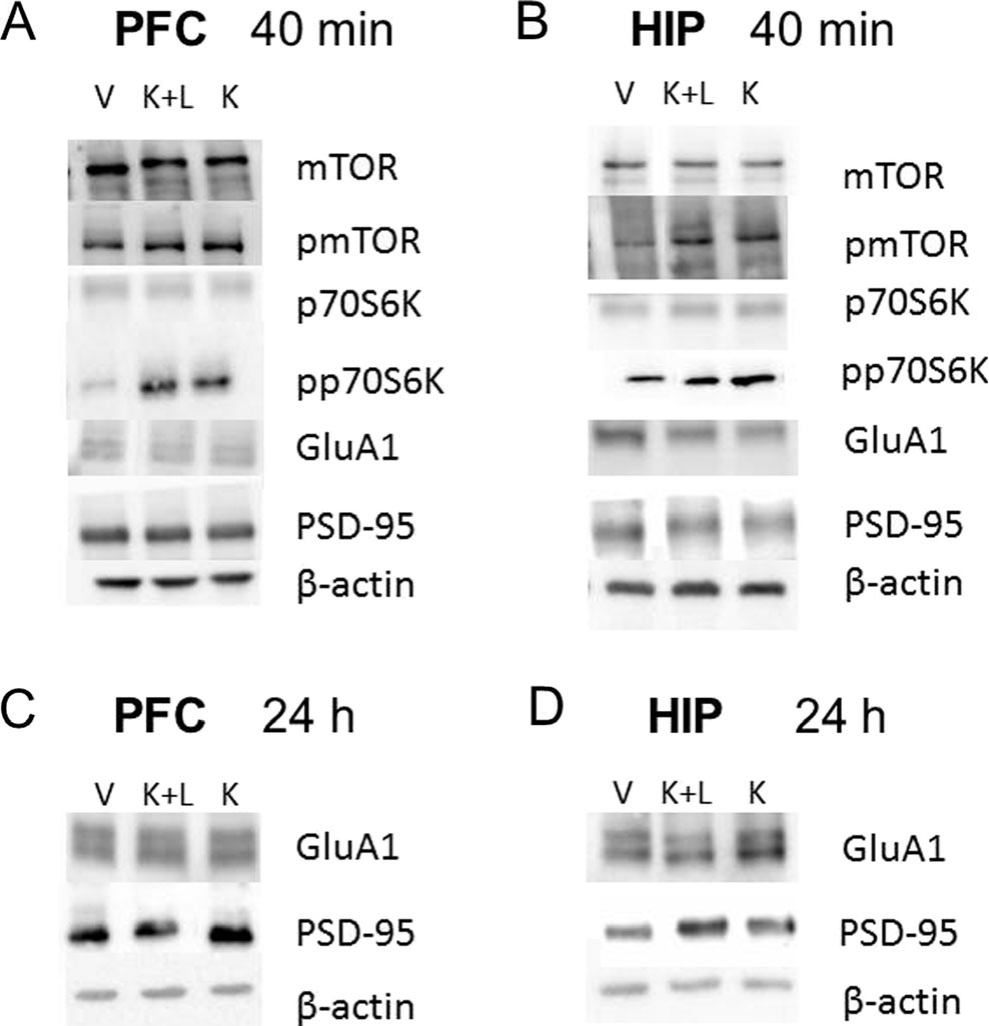
**a** Exemplary immunoblots of mTOR, pmTOR, p70S6K, pp70S6K, GluA1, PSD-95, and β-actin from the PFC of vehicle-treated group (*V*), ketamine (3 mg/kg) + LY341495 (0.3 mg/kg)-treated group (*K + L*), and ketamine (10 mg/kg)-treated group (*K*). The tissue was collected 40 min after drugs administration. **b** Exemplary immunoblots of mTOR, pmTOR, p70S6K, pp70S6K, GluA1, PSD-95, and β-actin from hippocampus of vehicle-treated group (*V*), ketamine (3 mg/kg) + LY341495 (0.3 mg/kg)-treated group (*K + L*), and ketamine (10 mg/kg)-treated group (*K*). The tissue was collected 40 min after drugs administration. **c** Exemplary immunoblots of GluR1, PSD95, and β-actin from the PFC of vehicle-treated group (*V*), ketamine (3 mg/kg) + LY341495 (0.3 mg/kg)-treated group (*K + L*), and ketamine (10 mg/kg)-treated group (*K*) The tissue was collected 24 h after drug administration. **d** Exemplary immunoblots of GluR1, PSD95, and β-actin from hippocampus of vehicle-treated group (*VEH*), ketamine (3 mg/kg) + LY341495 (0.3 mg/kg)-treated group (*KET + LY*), and ketamine (10 mg/kg)-treated group (*KET 10*). The tissue was collected 24 h after drug administration

**Fig. 12:**
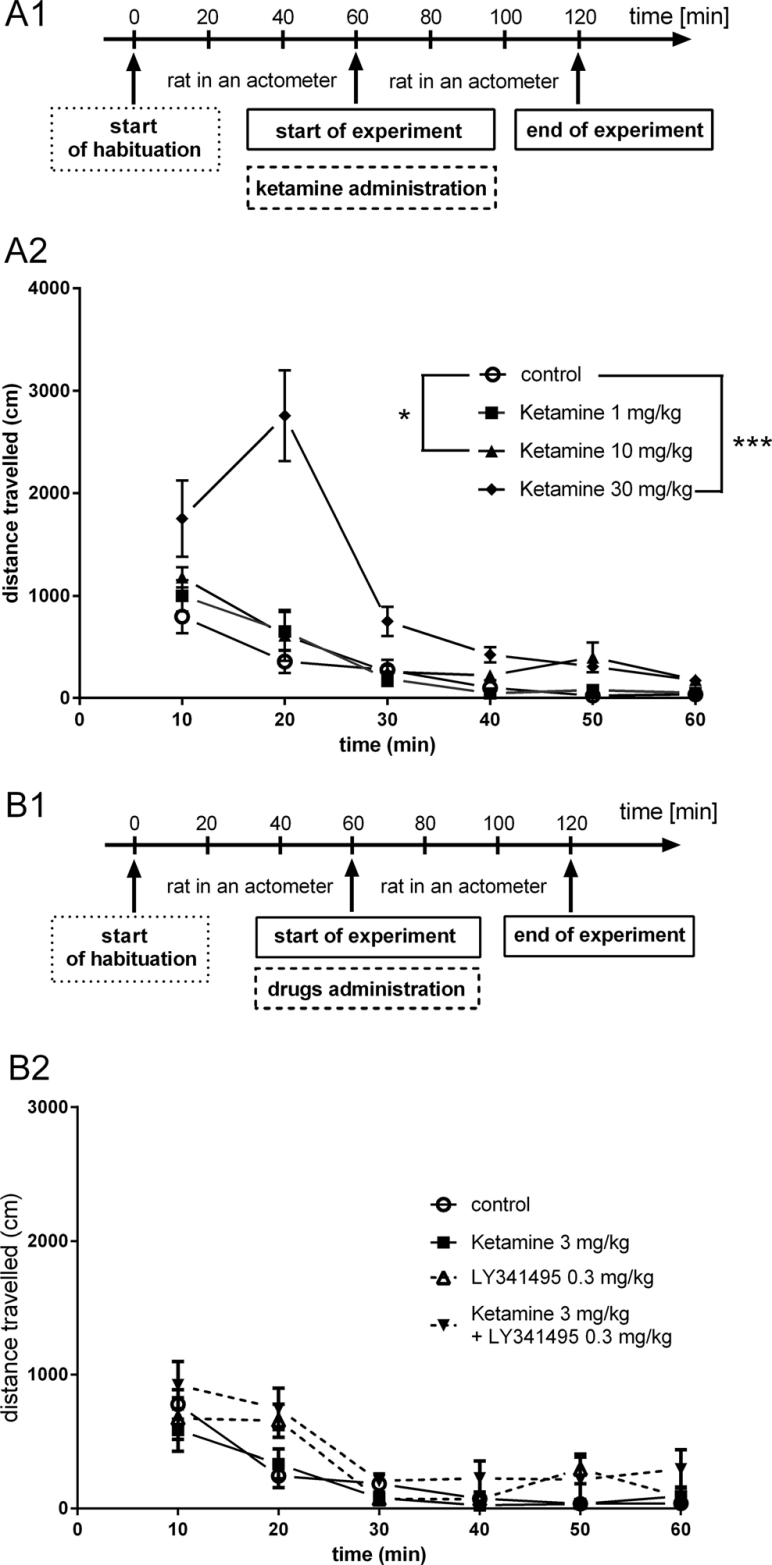
The effect of ketamine given separately (**a**) or jointly with LY341495 (**b**) on the locomotor activity of rats during a 60-min experimental session. Measurements started immediately after IP injections of tested substances. A1 and B1 represent schedules of the experimental procedures. A2 and B2 show the respective results. Values are expressed as the means ± SEM and were evaluated by repeated-measures ANOVA. *p < 0.05; ***p < 0.001 vs. control group

In rats previously acclimatized to actometers for 60 min, ketamine, at doses of 10 and 30 mg/kg, IP induced a rapid increase in the locomotor activity ([*F*(1, 14) = 6.799, *p* < 0.05] and [*F*(1, 14) = 26.4, *p* < 0.001], respectively) (Fig. 11). When used at a dose of 30 mg/kg, the effect of ketamine-induced hyperactivity reached a peak 20 min after injection and then gradually decreased (Fig. 12a). Next, we aimed to investigate whether a combination of ketamine and LY341495 doses, which had previously resulted in a positive effect in the FST, can induce behavioral effects in the ketamine-induced hyperlocomotion test. We found that ketamine (3 mg/kg) administered separately or in a combination with LY341495 (0.3 mg/kg) did not induce any effect in this test ([*F*(1, 14) = 1.133, *p* > 0.05] and [*F*(1, 14) = 1.03, *p* > 0.05], respectively). LY341495 (0.3 mg/kg), administered separately, also did not produce any hyperlocomotion, compared to control rats [*F*(1, 14) = 0.356, *p* > 0.05] (Fig. 12b).

### Ketamine-induced motor coordination impairment

**Fig. 13:**
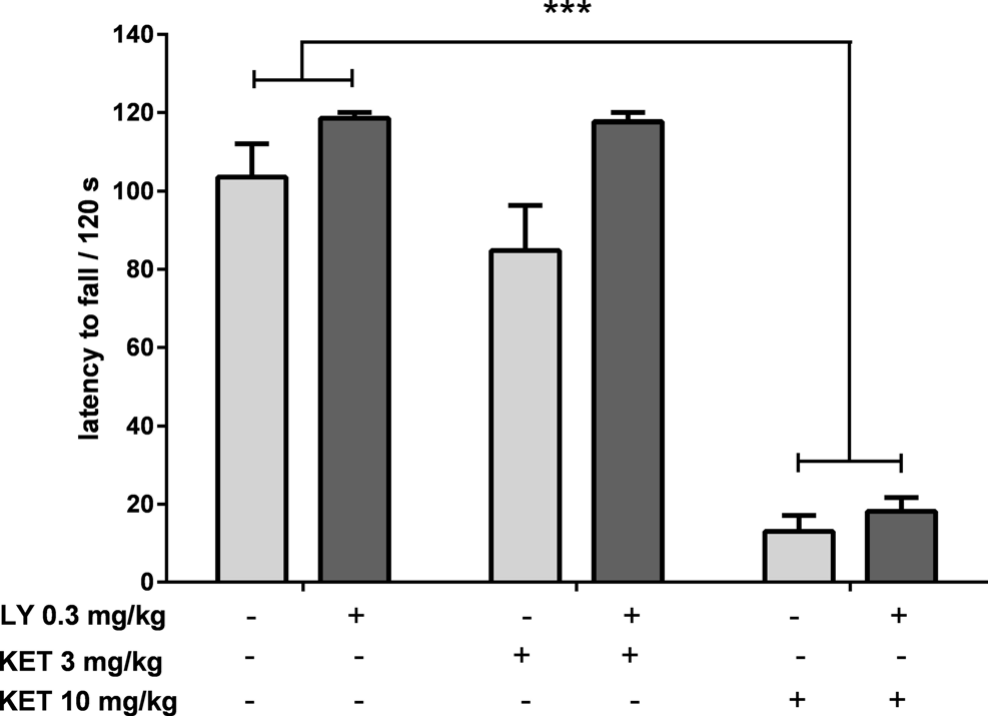
The effect of ketamine given separately or jointly with LY341495 on the latency to fall during the rota-rod test in rats. Values are expressed as the means ± SEM and were analyzed by two-way ANOVA. ***p < 0.0001 vs. vehicle

To study the effect of a combination of ketamine and LY341495, used at doses that had previously resulted in a positive effect in the FST, on motor coordination, four experimental groups were formed: a control group, ketamine (3 mg/kg) and LY341495 (0.3 mg/kg) groups, and a group given a mixture of the two. A two-way ANOVA showed lack of interaction between ketamine (3 mg/kg) and LY341495 (0.3 mg/kg) [*F*(1, 30) = 1.112; *p* > 0.05; Fig. 13], suggesting that ketamine action was not enhanced by LY341495 in this test. Furthermore, the results suggested an opposite tendency, i.e., the motor coordination of ketamine (3 mg/kg)-treated rats seemed to be improved by LY341495 (0.3 mg/kg) pretreatment. To deeply analyze this problem, a higher dose of ketamine was used in the experiment (10 mg/kg). A two-way ANOVA revealed that ketamine (10 mg/kg) significantly reduced the latency to fall from the rotating rod [*F*(1, 30) = 233; *p* < 0.0001; Fig. 13] and the pretreatment with LY341495 (0.3 mg/kg) did not change this effect (a lack of interaction between ketamine (10 mg/kg) and LY341495 (0.3 mg/kg) was found [*F*(1, 30) = 0.614; *p* < 0.05; Fig. 13]). The number of rats that fell off from the rotating rod during a 2-min experimental session was also recorded. In the group of control rats, 3 out of 11 animals (27 %) fell off of the rod, while in the groups of animals treated with ketamine (10 mg/kg) or ketamine (10 mg/kg) plus LY341495 (0.3 mg/kg), 100 % of the rats (*n* = 8) fell off during the same experimental period. The percentages of rats that fell from the rotating rod in the remaining experimental groups were 56 % in the group administered a 3-mg/kg dose of ketamine, 27 % in the group treated with LY341495 (0.3 mg/kg), and 27 % in the group treated with the combination of the two drugs.

## Discussion

The objective of our study was to investigate whether a group II mGlu receptor antagonist, LY341495, enhances the rapid and sustained antidepressant-like effects of ketamine. The FST in rats was chosen for this study, as this test is one of the most widely used in animal models for estimating antidepressant-like activity of new potential ADs (Porsolt et al. 1978; Cryan et al. 2005). Here, we applied a modified version of the FST, originally introduced by Detke et al. (1995), in which three specific types of behavior were measured: immobility, climbing, and swimming. Previous studies suggest that climbing is sensitive to catecholamine-based ADs, whereas swimming is modified by ADs, acting via modulation of the serotonergic system, including selective serotonin reuptake inhibitors (SSRIs) (Detke et al. 1995; Page et al. 1999). To summarize, the time of immobility is the main parameter that indicates the antidepressant activity of a tested compound, while the times of climbing and swimming only suggest the possible mechanisms of the compound’s action. Two time schedules were used in the current study, 40 min and 24 h after single injection of a drug, which reflect the rapid and prolonged effect, respectively. The first aim of this study was to determine the dose–effect curves for ketamine and LY341495 to identify the effective and sub-effective doses of both compounds. Dose-dependent antidepressant-like effects of ketamine and LY341495 have been previously reported using several rodent models of depression, including the FST (Chaki et al. 2004; Dwyer et al. 2012; Garcia et al. 2008, Gigliucci et al. 2013; Maeng et al. 2008; Koike et al. 2011; Koike and Chaki 2014; Li et al. 2010; Pałucha-Poniewiera et al. 2010).

In the FST, the antidepressant-like effect of classic ADs (including the SSRIs, MAO inhibitors, and tricyclics) is rapid and appears after short-term administration (three injections, 24, 5, and 1 h before the test) or even after single injection (usually 30–60 min before the test) (Borsini and Meli 1988). However, the therapeutic effect in depressive patients appears after several weeks of treatment. Thus, a dose-dependent antidepressant-like effect of ketamine and LY341495, which was well noticeable 40 min after single IP administration, only suggests the antidepressant activity of these drugs but does not suggest anything about the expected rate of emergence of a therapeutic effect in patients. Interestingly, both ketamine and LY341495 induced a significant and dose-dependent increase in the time of swimming, suggesting the involvement of the serotonergic system in this action. These results confirm the study by Gigliucci et al. (2013), who reported that ketamine induces sustained antidepressant-like activity via a serotonin-dependent mechanism. The results of our study also indicate that ketamine at the highest tested dose of 30 mg/kg affected climbing, indicating that the mechanism of its antidepressant activity also involves modulation of noradrenergic and/or dopaminergic systems. Based on the results obtained during determination of the dose–effect curves, sub-effective doses of both substances (3 mg/kg for ketamine and 0.1 mg/kg for LY341495) were chosen for joint administration. This drug combination induced strong antidepressant-like effects in the FST; however, a two-way ANOVA did not find any significant interaction between the tested drugs, indicating a lack of statistical significance. Because we observed a tendency for ketamine to decrease immobility time in this experiment, we decided to use an even lower dose of this drug and combine it with LY341495. At a dose of 1 mg/kg, ketamine did not change any behavioral parameter in the FST; however, ketamine significantly decreased immobility time when combined with LY341495 (0.1 mg/kg). These results clearly show that LY341495 enhances the rapid antidepressant activity of ketamine in the FST.

A separate set of experiments were performed in order to analyze the sustained antidepressant effects of the tested compounds. As previously mentioned, the prolonged antidepressant activity of ketamine and LY341495 in animal models of depression, including the FST, has been proposed to be related to functional synaptic changes in the PFC, which appear 24 h after single administration of the drug (Dwyer et al. 2012; Gigliucci et al. 2013; Li et al. 2010; Koike et al. 2011; Koike and Chaki 2014; Pałucha-Poniewiera et al. 2014). It is worth mentioning that other dosages proposed by other authors did not allow for the observation of the effectiveness of this drug (Bechtholt-Gompf et al. 2011; Popik et al. 2008; Yilmaz et al. 2002). Therefore, we applied a 24-h time lag between drug administration and behavioral testing, as a model of a sustained antidepressant effect of the tested compounds.

In the current study, we have shown that ketamine administered 24 h before the FST induced an antidepressant-like effect at only one tested dose, 10 mg/kg. Both lower (3 mg/kg) and higher (30 and 50 mg/kg) doses of ketamine were inactive in this test, although a marked tendency was observed that showed reduced immobility time after a dose of 30 mg/kg. It is interesting that similar effects of ketamine in the FST 24 h after injection have been published previously by Li et al. (2010), who showed that ketamine induced a strong antidepressant-like effect at a dose of 10 mg/kg, while it was completely inactive at a dose of 80 mg/kg.

The second drug used in this study, LY341495, induced a strong antidepressant-like effect in the FST when administered 24 h before the test at a dose of 1 mg/kg, which changed two behavioral parameters, immobility and swimming. At a dose of 0.3 mg/kg, LY341495 did not induce any effect. Based on the dose–response curves, the doses of 3 mg/kg for ketamine and 0.3 mg/kg for LY341495 were chosen as sub-effective and were injected jointly 24 h before the FST in order to investigate the potential antidepressant-like activity of this combination. The results have shown that joint administration of the tested compounds induced a significant decrease in the immobility time, thus indicating an antidepressant-like effect.

Compounds that increase locomotor activity can provide false positive results in the FST. Thus, another experiment in this study was designed to investigate the effect of the tested compounds, used at doses and schedules of applications previously applied in the FST, on the spontaneous locomotor activity of rats. We did not observe any changes in the spontaneous locomotor activity in rats, both 40 min and 24 h after administration of the tested doses of ketamine or LY341495, given individually or in combination. Thus, we conclude that the behavioral effects observed in the FST did not result from a general hyperactivity of rats but reflected the antidepressant activity of the tested compounds.

To investigate the possible mechanism of a sustained antidepressant-like effect of a combined administration of ketamine (3 mg/kg) and LY341495 (0.3 mg/kg), we analyzed the influence of this combination on the mTOR pathway and synaptic protein level in two brain areas, possibly involved in this action: the PFC and hippocampus. It has been established that activated mTOR phosphorylates the p70-kDa ribosomal protein S6 (p70S6K), which in turn induces the phosphorylation of the eukaryotic initiation factor (eIF4B), leading to the initiation of protein translation (Crino 2011). Thus, phosphorylation of mTOR and its downstream substrate, p70S6K, constitute robust biomarkers for the activation of the mTOR signaling pathway. Numerous data have indicated that ketamine, used at a dose that induces a sustained antidepressant-like effect (10 mg/kg), transiently activates mTOR signaling in rats. Such effect has been observed in the PFC 30 min to 1 h after ketamine administration (Li et al., 2010; Zhou et al., 2014) and in hippocampus 30 min after ketamine administration (Yang et al., 2013; Zhou et al., 2014). It has been proposed that ketamine-induced activation of mTOR pathway might be a crucial mechanism involved in the sustained antidepressant effects of ketamine (Li et al., 2010, Duman et al., 2012). Therefore, in this study, ketamine (10 mg/kg) has been used as a positive reference control. Since the similar activation of mTOR pathway has been observed in the rat PFC 1 h after single injection of LY341495, used at doses that produce a sustained antidepressant-like effect (Dwyer et al., 2012), we assumed that the activation of mTOR cascade and a subsequent increase in the level of synaptic proteins might be a mechanism possibly involved in the sustained antidepressant-like activity of a mixture of sub-effective doses of ketamine and LY341495 used in the study. Here we show that combined administration of ketamine and LY341495, at doses that induce a sustained antidepressant-like effect, produced a significant increase in the phosphorylation of both mTOR and p70S6K in the PFC and a noticeable tendency to increase these factors in the hippocampus of rats, indicating the involvement of mTOR pathway in the antidepressant-like effect of a mixture of ketamine and LY341495. The lack of statistical significance in hippocampus may partially result from a large scatter of results between individual rats in this group. Expression of selected synaptic proteins (GluA1 and PSD-95) has been also analyzed in the PFCs and hippocampi of rats administered with the mixture of ketamine (3 mg/kg) and LY341495 (0.3 mg/kg), and in a group of rats injected with solely ketamine (10 mg/kg), used as a reference control. We have found no changes in the level of synaptic proteins in hippocampus or the PFC, 40 min after IP injection of tested drugs. These results are in agreement with previously published data showing that ketamine, at a dose of 10 mg/kg, induced no changes in the expression level of synaptic proteins 30 min after injection and in the case of most proteins also 1 h after administration (Li et al., 2010). On the other hand, 24 h after treatment, we observed a significant increase in the protein level of GluA1 in the hippocampus and in the level of PSD-95 in the PFC and hippocampus, in rats administered with ketamine 10 mg/kg, thus partially confirming previous results of other authors (Li et al. 2010; Dwyer et al., 2012). A significant discrepancy between our data and the results of the Duman’s group is a lack of effect of ketamine on the level of GluA1 protein in the PFC, which we observed also in a repeat experiment (data not shown). Some methodological differences may account for these discrepancies. Importantly, a mixture of ketamine (3 mg/kg) and LY341495 (0.3 mg/kg), which was shown to induce a sustained antidepressant-like effect, similar to ketamine (10 mg/kg) did not influence expression level of any tested synaptic protein in the PFC but it induced a strong increase in the expression level of PSD-95 in the hippocampus, without affecting GluA1 level in this brain structure. On the basis of these results, we conclude that although a joint administration of LY341495 and ketamine, at doses that induce a sustained antidepressant-like effects in the FST, induces a rapid and robust activation of mTOR pathway in the PFC, it does not produce any increase in the level of any tested synaptic protein in this brain area. Thus, the enhanced level of GluA1 or PSD-95 in the PFC does not seem to be involved in the sustained antidepressant-like effect of a combined administration of sub-effective doses of tested compounds used in this study. On the other hand, it seems that in hippocampus, both jointly given ketamine and LY341495 as well as solely ketamine, used as a reference control (10 mg/kg), induce weaker effects on the mTOR pathway activity. However, a significant increase in the level of both tested synaptic proteins (GluA1 and PSD-95) has been observed 24 h after ketamine treatment and in the case of PSD-95 also after the combined administration of ketamine and LY341495. Therefore, it seems that the increased level of expression of PSD-95 protein hippocampus might be involved in the mechanism of antidepressant-like activity of the mixture of ketamine and LY341495 used in the study. Explanation of the mechanism of the antidepressant effects of sub-effective doses of ketamine and LY341495 requires further investigation.

In the last stage of this study, we focused on the side effect profile of the tested compounds. Systemic administration of the noncompetitive NMDA antagonists, such as MK-801, phencyclidine (PCP), and ketamine, to rodents causes a complex behavioral response called PCP-like effects, which include enhanced locomotion, body rolling, sniffing, and disturbances of motor coordination (Carter 1995). For our study, we choose two behavioral tests that may be considered as models of undesirable effects caused by ketamine. First, we used the ketamine-induced hyperlocomotion test. Ketamine and other NMDA antagonist, including MK-801, cause rapid hyperactivity in rats (Andine et al. 1999). It is believed that this effect resembles the attention deficit hyperactivity observed in humans after taking psychostimulants. Because antipsychotics selectively reduce this behavior, the hyperactivity induced by MK-801 is widely used as a model of positive symptoms of schizophrenia (Andine et al. 1999). Here, we observed significant hyperactivity induced by 30 mg/kg ketamine that peaked 20 min after ketamine administration in rats previously adapted to an actometer for 1 h. A smaller but still statistically significant effect of hyperactivity was induced by ketamine at a dose of 10 mg/kg. Such an activity profile confirms earlier studies on ketamine-induced hyperlocomotion (Gilmour et al. 2009). Subsequent studies showed a lack of an effect of lower doses of ketamine (1–3 mg/kg), LY341495 (0.3 mg/kg), or a combination of ketamine (3 mg/kg) with LY341495 (0.3 mg/kg) on the hyperactivity of rats. Thus, it seems that the combined administration of ketamine and LY341495, at doses that previously induced antidepressant-like effect in the FST, did not produce psychotomimetic-like effects in the ketamine-induced hyperlocomotion test. The lack of adverse effects of this drug combination is also suggested by the results of the rota-rod test, which is used as standard test for estimating motor coordination. Ketamine at a dose of 3 mg/kg, injected separately or in combination with LY341495 (0.3 mg/kg), did not significantly change the motor coordination of rats. At the same time, ketamine at a higher dose of 10 mg/kg strongly disturbed the motor coordination of all tested rats in this group, decreasing the latency to fall from the rota-rod by c.a. 90 %. This effect was not changed by LY341495 (0.3 mg/kg) pretreatment.

In conclusion, in this study, we found that the group II mGlu receptor antagonist LY341495 enhances the antidepressant-like effect of ketamine in the behavioral despair model of depression, indicating that the therapeutic dose of ketamine could be markedly reduced by its co-administration with LY341495 (a mGlu2/3 antagonist). Joint administration of these drugs might be a noteworthy alternative to the use of ketamine alone, which has dangerous consequences, such as addiction, when given at higher doses. Furthermore, our results encourage further examination of group II mGlu antagonists in humans, because its action seems to be similar to that of ketamine, but it lacks the adverse effects of noncompetitive NMDA receptor antagonists.
